# Automated segmentation of brain metastases with deep learning: A multi-center, randomized crossover, multi-reader evaluation study

**DOI:** 10.1093/neuonc/noae113

**Published:** 2024-07-11

**Authors:** Xiao Luo, Yadi Yang, Shaohan Yin, Hui Li, Ying Shao, Dechun Zheng, Xinchun Li, Jianpeng Li, Weixiong Fan, Jing Li, Xiaohua Ban, Shanshan Lian, Yun Zhang, Qiuxia Yang, Weijing Zhang, Cheng Zhang, Lidi Ma, Yingwei Luo, Fan Zhou, Shiyuan Wang, Cuiping Lin, Jiao Li, Ma Luo, Jianxun He, Guixiao Xu, Yaozong Gao, Dinggang Shen, Ying Sun, Yonggao Mou, Rong Zhang, Chuanmiao Xie

**Affiliations:** State Key Laboratory of Oncology in South China, Guangdong Provincial Clinical Research Center for Cancer, Sun Yat-sen University Cancer Center, Guang zhou, Guangdong Province, China; Department of Radiology, Sun Yat-sen University Cancer Center, Guang zhou, Guangdong Province, China; State Key Laboratory of Oncology in South China, Guangdong Provincial Clinical Research Center for Cancer, Sun Yat-sen University Cancer Center, Guang zhou, Guangdong Province, China; Department of Radiology, Sun Yat-sen University Cancer Center, Guang zhou, Guangdong Province, China; State Key Laboratory of Oncology in South China, Guangdong Provincial Clinical Research Center for Cancer, Sun Yat-sen University Cancer Center, Guang zhou, Guangdong Province, China; Department of Radiology, Sun Yat-sen University Cancer Center, Guang zhou, Guangdong Province, China; State Key Laboratory of Oncology in South China, Guangdong Provincial Clinical Research Center for Cancer, Sun Yat-sen University Cancer Center, Guang zhou, Guangdong Province, China; Department of Radiology, Sun Yat-sen University Cancer Center, Guang zhou, Guangdong Province, China; R&D Department, Shanghai United Imaging Intelligence Co., Ltd, Shanghai, China; Department of Radiology, Fujian Cancer Hospital, Fujian Medical University Cancer Hospital, Fuzhou, Fujian Province, China; Department of Radiology, The First Affiliated Hospital of Guangzhou Medical University, Guang zhou, Guangdong Province, China; Department of Radiology, Affiliated Dongguan Hospital, Southern Medical University, Dongguan, Guangdong Province, China; Department of Magnetic Resonance, Guangdong Provincial Key Laboratory of Precision Medicine and Clinical Translational Research of Hakka Population, Meizhou People’s Hospital, Meizhou, Guangdong Province, China; State Key Laboratory of Oncology in South China, Guangdong Provincial Clinical Research Center for Cancer, Sun Yat-sen University Cancer Center, Guang zhou, Guangdong Province, China; Department of Radiology, Sun Yat-sen University Cancer Center, Guang zhou, Guangdong Province, China; State Key Laboratory of Oncology in South China, Guangdong Provincial Clinical Research Center for Cancer, Sun Yat-sen University Cancer Center, Guang zhou, Guangdong Province, China; Department of Radiology, Sun Yat-sen University Cancer Center, Guang zhou, Guangdong Province, China; State Key Laboratory of Oncology in South China, Guangdong Provincial Clinical Research Center for Cancer, Sun Yat-sen University Cancer Center, Guang zhou, Guangdong Province, China; Department of Radiology, Sun Yat-sen University Cancer Center, Guang zhou, Guangdong Province, China; State Key Laboratory of Oncology in South China, Guangdong Provincial Clinical Research Center for Cancer, Sun Yat-sen University Cancer Center, Guang zhou, Guangdong Province, China; Department of Radiology, Sun Yat-sen University Cancer Center, Guang zhou, Guangdong Province, China; State Key Laboratory of Oncology in South China, Guangdong Provincial Clinical Research Center for Cancer, Sun Yat-sen University Cancer Center, Guang zhou, Guangdong Province, China; Department of Radiology, Sun Yat-sen University Cancer Center, Guang zhou, Guangdong Province, China; State Key Laboratory of Oncology in South China, Guangdong Provincial Clinical Research Center for Cancer, Sun Yat-sen University Cancer Center, Guang zhou, Guangdong Province, China; Department of Radiology, Sun Yat-sen University Cancer Center, Guang zhou, Guangdong Province, China; State Key Laboratory of Oncology in South China, Guangdong Provincial Clinical Research Center for Cancer, Sun Yat-sen University Cancer Center, Guang zhou, Guangdong Province, China; Department of Radiology, Sun Yat-sen University Cancer Center, Guang zhou, Guangdong Province, China; State Key Laboratory of Oncology in South China, Guangdong Provincial Clinical Research Center for Cancer, Sun Yat-sen University Cancer Center, Guang zhou, Guangdong Province, China; Department of Radiology, Sun Yat-sen University Cancer Center, Guang zhou, Guangdong Province, China; State Key Laboratory of Oncology in South China, Guangdong Provincial Clinical Research Center for Cancer, Sun Yat-sen University Cancer Center, Guang zhou, Guangdong Province, China; Department of Radiology, Sun Yat-sen University Cancer Center, Guang zhou, Guangdong Province, China; State Key Laboratory of Oncology in South China, Guangdong Provincial Clinical Research Center for Cancer, Sun Yat-sen University Cancer Center, Guang zhou, Guangdong Province, China; Department of Radiology, Sun Yat-sen University Cancer Center, Guang zhou, Guangdong Province, China; State Key Laboratory of Oncology in South China, Guangdong Provincial Clinical Research Center for Cancer, Sun Yat-sen University Cancer Center, Guang zhou, Guangdong Province, China; Department of Radiology, Sun Yat-sen University Cancer Center, Guang zhou, Guangdong Province, China; State Key Laboratory of Oncology in South China, Guangdong Provincial Clinical Research Center for Cancer, Sun Yat-sen University Cancer Center, Guang zhou, Guangdong Province, China; Department of Radiology, Sun Yat-sen University Cancer Center, Guang zhou, Guangdong Province, China; State Key Laboratory of Oncology in South China, Guangdong Provincial Clinical Research Center for Cancer, Sun Yat-sen University Cancer Center, Guang zhou, Guangdong Province, China; Department of Radiology, Sun Yat-sen University Cancer Center, Guang zhou, Guangdong Province, China; State Key Laboratory of Oncology in South China, Guangdong Provincial Clinical Research Center for Cancer, Sun Yat-sen University Cancer Center, Guang zhou, Guangdong Province, China; Department of Radiology, Sun Yat-sen University Cancer Center, Guang zhou, Guangdong Province, China; Department of Radiology, The First Affiliated Hospital of Guangzhou Medical University, Guang zhou, Guangdong Province, China; State Key Laboratory of Oncology in South China, Guangdong Provincial Clinical Research Center for Cancer, Sun Yat-sen University Cancer Center, Guang zhou, Guangdong Province, China; R&D Department, Shanghai United Imaging Intelligence Co., Ltd, Shanghai, China; R&D Department, Shanghai United Imaging Intelligence Co., Ltd, Shanghai, China; Department of Radiation Oncology, Sun Yat-Sen University Cancer Center, Guang zhou, Guangdong Province, China; Department of Neurosurgery, Sun Yat-Sen University Cancer Center, Guang zhou, Guangdong Province, China; State Key Laboratory of Oncology in South China, Guangdong Provincial Clinical Research Center for Cancer, Sun Yat-sen University Cancer Center, Guang zhou, Guangdong Province, China; Department of Radiology, Sun Yat-sen University Cancer Center, Guang zhou, Guangdong Province, China; State Key Laboratory of Oncology in South China, Guangdong Provincial Clinical Research Center for Cancer, Sun Yat-sen University Cancer Center, Guang zhou, Guangdong Province, China; Department of Radiology, Sun Yat-sen University Cancer Center, Guang zhou, Guangdong Province, China

**Keywords:** automatic segmentation, brain metastases, deep learning, MRI, multi-reader multi-case

## Abstract

**Background:**

Artificial intelligence has been proposed for brain metastasis (BM) segmentation but it has not been fully clinically validated. The aim of this study was to develop and evaluate a system for BM segmentation.

**Methods:**

A deep-learning-based BM segmentation system (BMSS) was developed using contrast-enhanced MR images from 488 patients with 10338 brain metastases. A randomized crossover, multi-reader study was then conducted to evaluate the performance of the BMSS for BM segmentation using data prospectively collected from 50 patients with 203 metastases at 5 centers. Five radiology residents and 5 attending radiologists were randomly assigned to contour the same prospective set in assisted and unassisted modes. Aided and unaided Dice similarity coefficients (DSCs) and contouring times per lesion were compared.

**Results:**

The BMSS alone yielded a median DSC of 0.91 (95% confidence interval, 0.90–0.92) in the multi-center set and showed comparable performance between the internal and external sets (*P* = .67). With BMSS assistance, the readers increased the median DSC from 0.87 (0.87–0.88) to 0.92 (0.92–0.92) (*P* < .001) with a median time saving of 42% (40–45%) per lesion. Resident readers showed a greater improvement than attending readers in contouring accuracy (improved median DSC, 0.05 [0.05–0.05] vs 0.03 [0.03–0.03]; *P* < .001), but a similar time reduction (reduced median time, 44% [40–47%] vs 40% [37–44%]; *P* = .92) with BMSS assistance.

**Conclusions:**

The BMSS can be optimally applied to improve the efficiency of brain metastasis delineation in clinical practice.

## Graphical Abstract

**Graphical Abstract:**
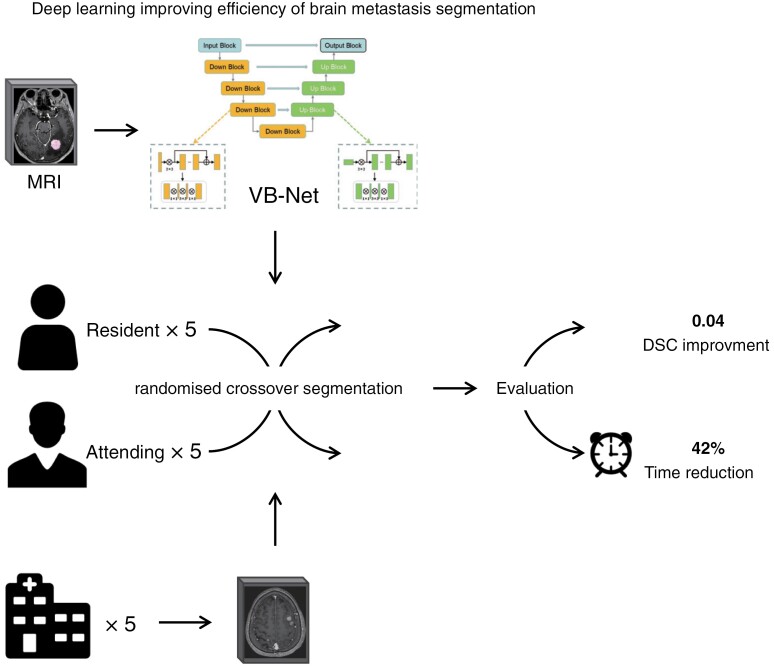

**Key Points**

• The BMSS had high and robust performance at brain metastasis segmentation on MR images.
• The BMSS improved radiologists’ efficiency at brain metastasis segmentation.

**Importance of the Study**

Stereotactic radiosurgery (SRS) is a validated treatment for brain metastases (BMs) but requires accurate tumor contouring, which is labor-intensive and prone to substantial operator variability. Artificial intelligence (AI) has been proposed for BM segmentation, but its generalizability and clinical value remain unclear. Here, we showed that a fully connected deep convolutional neural network system, developed using a large number of lesions (>10000 lesions), accurately and robustly segmented BMs in a test dataset prospectively collected from a multi-center patient cohort. Furthermore, we conducted a randomized crossover, multi-reader, multi-case study to evaluate the effect of AI assistance. In this reading experiment, we showed that AI assistance improved the delineating accuracy, with a median time saving of 42% for assisted readers. Our results indicate that this system can be used as an assistance tool in SRS for BMs.

The outcomes of patients with cancer have improved due to advances in local and systemic therapies,^1^ and thus, the incidence of brain metastases (BMs) has gradually increased, occurring in 20%–40% of patients with systemic cancer.^1,2^ Stereotactic radiosurgery (SRS) has played an increasingly essential role in the management of BM, even in patients with more than 30 lesions,^3^ and has become a validated treatment for BM.^4,5^ To effectively eradicate BM while minimizing damage to perilesional brain tissue, SRS requires meticulous delineation of the target tumor. This process is extremely labor-intensive and prone to high variability between practitioners.^6–8^

Artificial intelligence (AI) has been used for the detection and/or segmentation of BMs over the past decade.^9,10^ Of the state-of-the-art auto-contouring algorithms, deep convolutional neural networks (CNNs), particularly those with 3-dimensional U-Net and DeepMedic architecture,^10–15^ have emerged as powerful methods for segmentation. Such models dedicated to BMs have been reported in many preclinical studies, but most of these models have been trained and tested on a single-institution dataset, and almost no clinical trials have been conducted to determine how the results translate into clinical practice.^11–17^ Despite promising standalone AI performance in internal and preclinical evaluations, concerns remain about the robustness and generalizability of the models. Additionally, AI tools are likely to perform unexpectedly poorly in clinical settings, a concern known as the implementation gap.^18^ A robust AI tool for BM segmentation and a prospective study assessing its efficiency within the radiation therapy clinical workflow are warranted.

We aimed to develop a deep-learning brain metastasis segmentation system (BMSS) based on a large dataset and to provide clinical validation by conducting a randomized crossover, multi-reader, multi-case (MRMC) study using multi-center prospective data, for which multiple radiologists with different levels of experience performed target contouring. We evaluated the standalone performance of the BMSS and compared the readers’ contouring accuracy and time with and without AI assistance.

## Materials and Methods

### Study Design and Participants

**Figure 1.**
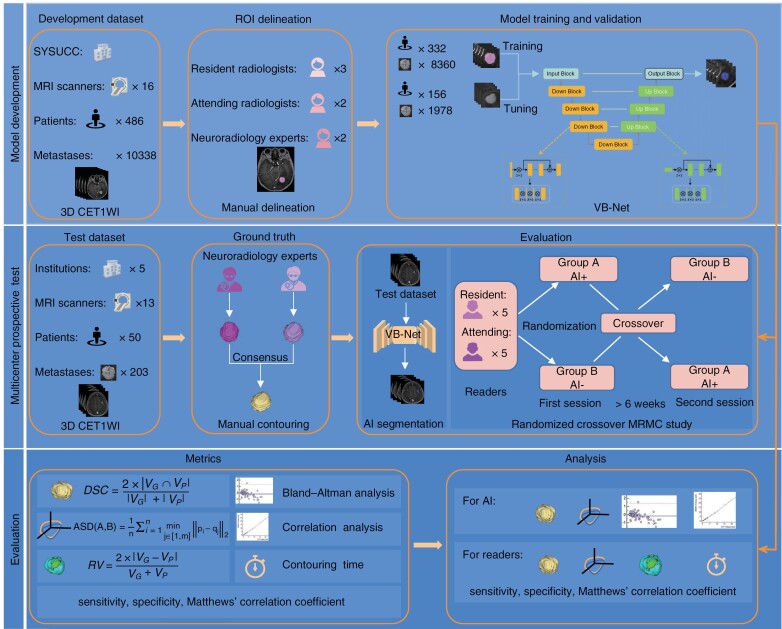
Study overview. VB-Net, a 3D segmentation model derived from V-Net, was trained and tuned on brain 3D CET1WI images collected from SYSUCC. After completing model development, test data from 5 medical centers were prospectively included to validate the model’s robustness. In addition, a randomized crossover reading experiment was designed to evaluate the effect of the developed system on brain metastasis contours for readers with various levels of experience. The performance of the network alone and of readers with and without assistance was assessed. SYSUCC, Sun Yat-sen University Cancer Center; 3D CET1WI, 3-dimensional contrast-enhanced T1-weighted imaging; ROI, region of interest; DSC, Dice similarity coefficient; ASD, average surface distance; RVD, relative volume difference; AI, artificial intelligence; MRMC, multi-reader, multi-case.

In this multi-center study, we collected 3-dimensional, contrast-enhanced T1-weighted imaging (3D CET1WI) data acquired at the Sun Yat-sen University Cancer Center (SYSUCC) from patients with newly diagnosed BM. These data were used to train and tune a fully connected VB-Net proposed by Han et al.^19^ for automated BM segmentation. To validate the robustness of the BMSS, prospective data from 5 medical centers across China were consecutively included. In addition, a randomized crossover MRMC study was conducted with multiple radiologists of varying experience to evaluate the potential effect of the BMSS in a clinical setting (Figure 1).

**Figure 2.**
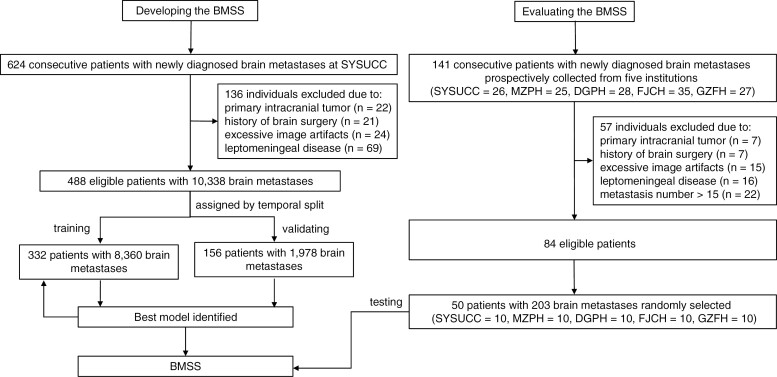
Study patients. Patients from SYSUCC with newly diagnosed brain metastases were consecutively included to develop the BMSS. Another prospective test dataset collected from 5 centers was used to evaluate the model’s generalizability and its effect on reader contouring. BMSS, brain metastasis segmentation system; SYSUCC, Sun Yat-sen University Cancer Center; MZPH, Meizhou People’s Hospital; DGPH, Dongguan People’s Hospital; FJCH, Fujian Cancer Hospital; GZFH, The First Affiliated Hospital of Guangzhou Medical University; n, number of patients.

The study patients are described in Figure 2. From August 15, 2019 to November 18, 2020, 624 consecutive patients who were newly diagnosed with BM and underwent 3D contrast-enhanced brain MRI at SYSUCC were retrospectively included as the development set. These data were then divided into training and validation datasets (2:1) with a temporal split on April 30, 2020. Once model tuning was complete, consecutive cohorts, defined as the test set, were prospectively recruited from SYSUCC, Meizhou People’s Hospital, Dongguan People’s Hospital, Fujian Cancer Hospital, and The First Affiliated Hospital of Guangzhou Medical University from February to August 2021. The inclusion criteria were the same for the development and test sets. We included patients (1) with extracranial primary tumor(s) confirmed by pathology; (2) with newly developed BM; and (3) who underwent 3D-enhanced brain MRI and at least one follow-up MRI. Only the initial scan with at least one lesion was assessed in this study. We excluded patients (1) with primary intracranial tumor(s); (2) with meningeal metastasis; (3) who had undergone brain surgery; or (4) with excessive artifacts in their images. There were no limitations on the size of the metastases. Given the application of SRS to patients with limited brain metastases and the reading workload in the MRMC study, patients in the test set with more than 15 BMs were excluded. The detailed distributions of all datasets are provided in Supplementary Materials Part 1.

Our study was approved by the Ethics Committee of SYSUCC (No. B2021-198-01) and was undertaken according to the principles of the Declaration of Helsinki. For patients who were retrospectively recruited, informed consent was exempted by the institutional review board. Informed consent was obtained from patients included in the prospective dataset.

### MRI Protocols

MRI data were acquired from 16 different scanners produced by 4 manufacturers, with various scanning protocols based on gradient echo sequences. The key acquisition parameters are provided in Supplementary Materials Part 2. All scans and clinical data were carefully reviewed by 2 board-certified neuroradiologists with 12 and 8 years of experience.

### Ground Truth

There were no overlapping patients among the training, validation, and test datasets. The ground truth (GT) masks for the development dataset were manually established slice by slice on the axial 3D CET1WI images. This task was performed by 5 radiologists with 3, 3, 4, 8, and 10 years of experience, using ITK SNAP (version 3.6; www.itksnap.org). The masks were refined and confirmed by 2 neuroradiologists with 12 and 8 years of experience.

For the test dataset, the GT was independently manually delineated by 2 neuroimaging experts with 13 and 15 years of experience. The experts had full access to the patients’ histories and previous imaging studies when they defined the GT. Three-dimensional reconstructions of the contours they generated were visualized simultaneously and compared between experts. The median inter-expert Dice similarity coefficient (DSC) reached 0.90 (95% confidence interval [CI], 0.89–0.91). Any divergences were resolved by consensus. The BM size was defined as the largest cross-sectional dimension of the GT on the axial image, and the volume was calculated based on the GT.

### Model Architecture and Implementation

We developed a BM-detection system based on a cascade feature pyramid network in our previous study.^20^ In the current study, the BMSS was an end-to-end 3D segmentation model derived from V-Net. Specifically, we integrated the detection architecture with the BMSS. The detected lesions were automatically segmented. V-Net was optimized and designed as VB-Net.^19^ The main goal was to apply model compression to reduce the extensive number of network parameters and allow the rapid training and deployment of the segmentation network. Specifically, we added a bottleneck structure to V-Net to form VB-Net, as shown in Figure 1. Each down-block and up-block was replaced with a bottleneck structure that included 3 convolutional layers. The first layer reduced the number of channels using a 1 × 1 × 1 convolution kernel, the second layer performed 3 × 3 × 3 spatial convolution, and the third layer applied a 1 × 1 × 1 convolution kernel to increase the number of channels to match the V-Net structure. Thus, the number of parameters was significantly reduced, and the experimental results showed that the new network performed as well as the original network. Finally, without loss of accuracy, the size of the VB-Net model was reduced by 96.5% compared with V-Net, which is suitable for many common purposes, including use on mobile devices. Additional details about the architecture are provided in Supplementary Materials Part 3.

Model implementation was based on the Pytorch framework, an open-source Python deep-learning library.^21^ We used focal loss (α = 2; β = 0.999) as the loss function. The model was trained using the Adam optimization algorithm to dynamically adjust the learning rate. The initial learning rate was 1 × 10^−4^ The training used an Intel® Xeon® CPU E5-2698 v4 @ 2.20 GHz central processing unit (Intel, Santa Clara) and an Nvidia Tesla V100-SXM2, 32G × 8 graphics processing unit with CUDA version 10.1 (Nvidia, Santa Clara). Additionally, data augmentation (eg, random shifting and rotation) was performed to enrich the training dataset.

### Clinical Evaluation

The reading was designed as a randomized crossover pattern, as shown in Figure 1. In April 2022, 10 independent readers with various levels of experience (including 2 in-training physicians [1 year of experience], 3 board-certified residents [2–3 years of experience], and 5 attending radiologists [5–10 years of experience]) working at SYSUCC were trained on the segmentation task with the BMSS using a small independent sample of cases that were not included in the development or test sets. None of the readers were involved in patient enrollment or image labeling.

From May to July 2022, the readings were performed on dedicated medical workstations displaying de-identified lossless 3D CET1WI images. All readers contoured all of the test cases with and without BMSS assistance. To reduce potential bias derived from memory and the read sequence, the readers were randomized into either assisted-first or unassisted-first segmentation sessions. The 2 contour modes were separated by a washout period of at least 6 weeks, along with ongoing full-time clinical work. During the second session, the same cases were presented in a random order, and the readers did not have access to clinical information, other readers’ contouring results, or the experts’ GT. To simulate a contouring situation similar to the normal clinical condition, the readers were not instructed to complete all cases in a single-contouring session, but were allowed to fulfill all tasks within 10 days. The contouring time was recorded for each lesion.

### Statistical Analysis

A sample size calculation for AI-based segmentation has not been established, and no consensus has been reached. The development cohort size in this study followed the common principle of deep-learning research, whereby more data tend to yield better results, while taking into account the effort of manually establishing the reference standard. The test sample size was calculated using the method provided in Supplementary materials Part 4.

The evaluation metrics were assessed as follows.^22,23^

(1) The amount of overlap at the intersection of the BMSS-predicted volume (PV) and GT volume (GV) was assessed using the DSC, defined as:


$$\begin{array}{l} DSC=\frac{2\left|PV\cap GV\right|}{\left|PV\right|+\left|GV\right|},\;\;\;0\leq DSC\leq 1. \end{array}$$

A DSC value of 1 indicates complete consistency, while a DSC value of 0 indicates no overlap.

(2) The difference between the PV and GV was determined using the relative volume difference (RVD), defined as:


$$\begin{array}{l} RVD=\frac{2\left|PV-GV\right|}{\left|PV\right|+\left|GV\right|}\times 100\;\%\;,0\leq RVD\leq 100\;\%\;. \end{array}$$

An RVD value of 100% indicates a complete difference, while an RVD value of 0 indicates no difference.

(3) The boundary discrepancies between segmented structures were measured using the average surface distance (ASD, mm), defined as:


$$\begin{array}{l} ASD\left(PV,GV\right)=\frac{1}{n}\underset{i=1}{\overset{n}{\sum }}\;\underset{j\in \left[1,m\right]}{\min}\;∥p_{i}-g_{j}{∥}_{2}. \end{array}$$

where *n* and *m* are the numbers of voxels in the PV and GV, respectively; *p*_*i*_ and *g*_*i*_ are the *i-*th and *j-*th boundary voxels of the PV and GV, respectively; and the inner minimum operator measures the surface distance of *p*_*i*_ to the GV.

(A) To compare quantitative volumetric agreements between experts’ manual and automatic segmentations, Spearman’s correlation was assessed and Bland–Altman plots were constructed.^24^
(B) To comprehensively quantify the performance of the BMSS, the patient-wise DSC_p_, voxel-wise sensitivity, specificity, and Matthews correlation coefficient (MCC) values of the readers were calculated (Supplementary Materials Part 4).

In comparison with the GV, the DSC, ASD, correlation analysis, and Bland–Altman analysis were computed for the BMSS. The DSC, ASD, RVD, sensitivity, specificity, MCC, and contouring time (per lesion and per patient) were calculated for the readers. The 95% CIs for these metrics were calculated using bootstrapping with 1000 iterations. The DSC values were compared between the internal and external test datasets using the Mann–Whitney *U* test. The DSC values for the BMSS for metastases of different volumes were compared using the Kruskal–Wallis *H* test. Wilcoxon matched-pairs signed-rank tests were used to compare the DSC, RVD, and contouring time between the 2 contouring modes for the readers. Bland–Altman analysis was performed using MedCalc Statistical Software (v15.2.0; MedCalc Software, Ostend, Belgium). Other statistical analyses were performed using SPSS (v25.0; IBM Corp., Armonk) and were 2-sided, with a significance level of .05.

## Results

**Figure 3.**
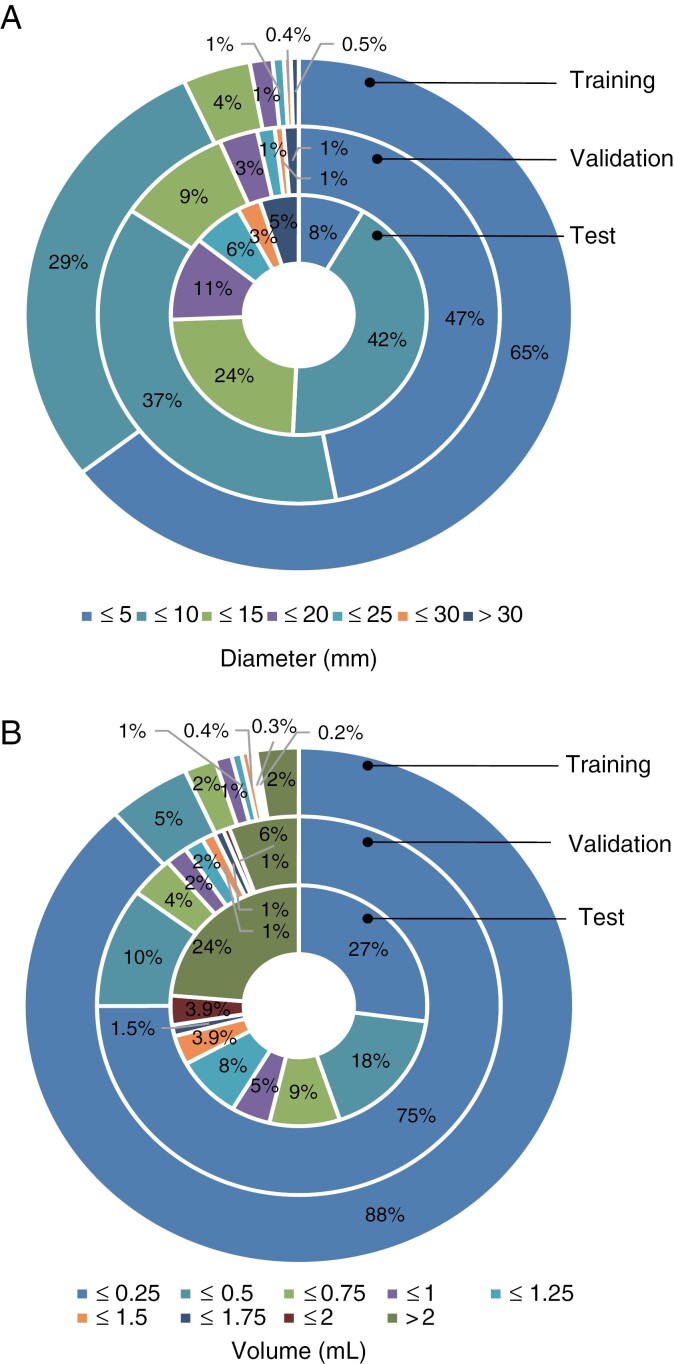
The size distribution of the datasets. (A) and (B) show the diameter and volume distributions of the brain metastases, respectively. The outer, middle, and inner rings represent the training set, validation set, and test set, respectively.

The complete study cohort consisted of 10541 BMs from 538 consecutive patients. The training, internal validation, and multi-center test datasets comprised 332 patients with 8360 BMs, 156 patients with 1978 BMs, and 50 patients with 203 BMs, respectively. The baseline characteristics of these patients and their metastases are shown in Table 1 and Figure 3. The details of the individuals excluded are provided in Supplementary materials Part 1.

**Table 1.** Patient Demographics and Brain Metastasis Characteristics

Characteristics	Training	Validation	Test						Total
			SYSUCC	MZPH	DGPH	FJCH	GZFH	Total	
Patients	332	156	10	10	10	10	10	50	538
Male	189 (57)	85 (55)	7 (70)	8 (80)	7 (70)	8 (80)	5 (50)	35 (70)	309 (57)
Age (years)	57 ± 11	56 ± 11	58 ± 12	64 ± 6	59 ± 7	59 ± 6	58 ± 10	59 ± 9	57 ± 11
Brain metastases									
Number	8,360	1,978	57	51	35	34	26	203	10,541
Size									
Diameter (mm)	5.3 ± 4.1	7.0 ± 5.6	13.6 ± 8	12.1 ± 6.9	10.7 ± 5.4	14.2 ± 8.2	14.0 ± 9.1	12.8 ± 7.7	5.8 ± 4.7
Volume (mL)	0.37 ± 3.1	0.72 ± 3.6	2.4 ± 4.8	2.0 ± 5.0	1.2 ± 2.5	2.4 ± 3.6	2.1 ± 3.4	2.0 ± 4.2	0.47 ± 3.2
MRI field (3.0 T)	304 (74)	124 (77)	8 (80)	9 (90)	10 (100)	10 (100)	8 (80)	45 (90)	474 (88)
Primary Tumors									
Lung cancer	303 (91)	128 (82)	5 (50)	9 (90)	8 (80)	8 (80)	9 (90)	39 (78)	470 (87)
Breast cancer	9 (3)	14 (9)	0	0	2 (20)	1 (10)	1 (10)	4 (8)	27 (5)
** **Melanoma	6 (2)	3 (2)	2 (20)	0	0	0	0	2 (2)	11 (2)
Colorectal cancer	4 (1)	2 (1)	1 (10)	1 (10)	0	1 (10)	0	3 (3)	7 (1)
Others	10 (3)	9 (6)	2 (20)	0	0	0	0	2 (2)	23 (4)

The data are presented as the number, number (percentage), or mean ± *SD*.

The training and validation datasets were collected from SYSUCC, and the external test datasets were collected from other 4 institutions.

SYSUCC, Sun Yat-sen University Cancer Center; MZPH, Meizhou People’s Hospital; DGPH, Dongguan People’s Hospital; FJCH, Fujian Cancer Hospital; GZFH, The First Affiliated Hospital of Guanzhou Medical; T, Tesla.

### AI Standalone Performance

**Figure 4.**
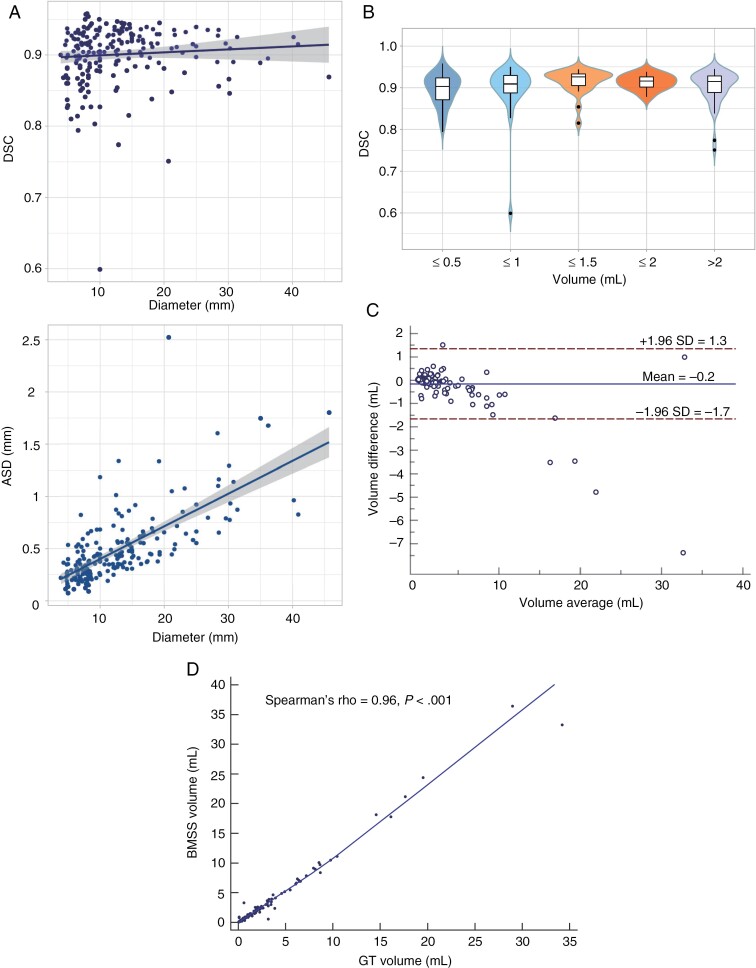
Performance of the BMSS on the test dataset. Overall, the BMSS alone achieved a median DSC of 0.91 (95% CI, 0.90–0.92) and a median ASD of 0.40 mm (95% CI, 0.36–0.44). The predicted volumes showed high agreement with the expert ground truth volumes. (A) Scatter plots of the DSC (upper) and ASD (lower), (B) DSC values of the model for different lesion sizes, (C) Bland–Altman plots, (D) Correlation analysis. BMSS, brain metastasis segmentation system; DSC, Dice similarity coefficient; ASD, average surface distance; GT, ground truth; rho, correlation coefficient.

The prospective test dataset yielded a median DSC of 0.91 (0.90–0.92) and a median ASD of 0.40 mm (0.36–0.44 mm; Figure 4A). The BMSS achieved similar accuracy among variable volumes (Kruskal–Wallis *H* test, *P* = .08; Figure 4B), accompanied by high agreement between the expert GVs, as depicted in Figure 4C and 4D. In subgroup analysis, the BMSS showed comparable performance between the internal SYSUCC test set and the external test set consisting of data from 4 centers (median DSC, 0.91 [0.90–0.92] vs 0.91 [0.90–0.92], *P* = 0.67; median ASD, 0.42 mm [0.35–0.53 mm] vs 0.39 mm [0.35–0.44 mm], *P* = .22).

### Effect of AI Assistance on the Readers’ Performance

**Figure 5.**
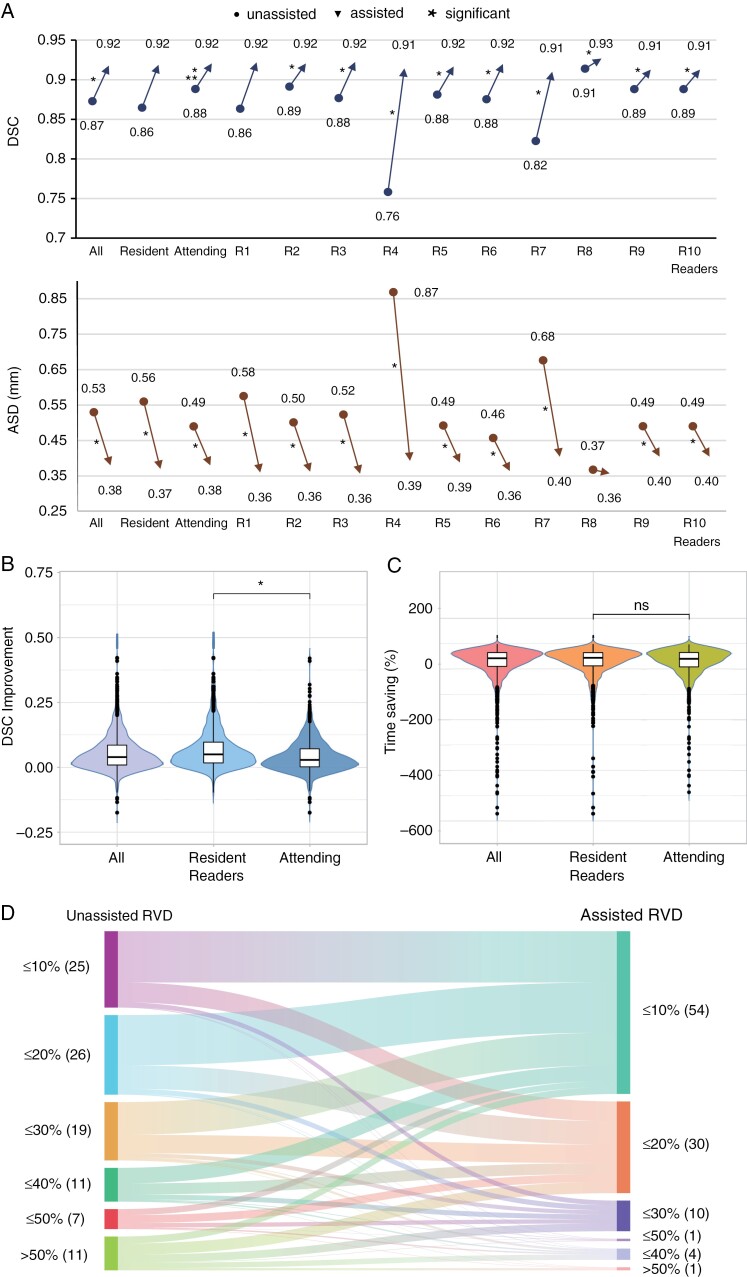
Performance of unassisted and assisted readers. (A) DSC (upper) and ASD (lower) values for readers in unassisted (●) and assisted (▲) modes; *, statistically significant difference; R1–5, resident radiologists and R6–10, attending radiologists. (B) DSC value improvement and (C) time saving for assisted readers with different levels of experience, ns, no significant difference, (D) RVD changes for readers without (left) and with (right) assistance. The data are presented as the RVD (percentage). DSC, Dice similarity coefficient; ASD, average surface distance; RVD, relative volume difference.

Algorithm assistance improved the contouring accuracy. The median increase in the DSC was 0.04 (0.04–0.04), with a median DSC of 0.87 (0.87–0.88) for the unassisted mode and 0.92 (0.92–0.92) for the assisted mode (*P *< .001). Figure 5A demonstrates the improvement in DSC (upper figure) and the reduction in ASD (lower figure) with AI assistance for each of the readers. When unassisted, the attending readers (designated as R6–R10) had a significantly higher contouring accuracy than the resident readers (R1–R5; median DSC, 0.86 [0.86–0.87] vs 0.88 [0.88–0.89]; *P* < .001). When using the BMSS, both groups performed similarly well (median DSC, 0.92 [0.92–0.92] vs 0.92 [0.91–0.92]; *P* = .39). In particular, the resident readers achieved a greater refinement of contouring accuracy than the attending readers (improved median DSC, 0.05 [0.05–0.05] vs 0.03 [0.03–0.03]; *P* < .001; Figure 5B). The order of the contouring mode (unassisted first vs assisted first) had a slight positive effect on contouring accuracy (median, 0.04 [0.03–0.04] vs 0.04 [0.04–0.05]; *P* = .02). At the patient level, the same trends were observed (unassisted vs assisted, all readers median DSC_*p*_, 0.87 [0.86–0.87] vs 0.91 [0.91–0.92], *P* < .001; resident median DSC_*p*_, 0.86 [0.85–0.87] vs 0.91 [0.91–0.91]; *P* < .001; attending median DSC_*p*_, 0.88 [0.87–0.89] vs 0.91 [0.91–0.92], *P* < .001; DSC*p* improvement, resident vs attending 0.048 [0.041–0.056] vs 0.029 [0.022–0.036], *P* < .001).

The median ASD was significantly lower with AI assistance than without assistance (0.38 mm [0.37–0.39 mm] vs 0.53 mm [0.52–0.54 mm]; *P *< .001). Similar to the DSC improvement, the reduction in the median ASD was greater for the resident radiologists than the attending radiologists (reduced median ASD, 0.18 mm [0.17–0.19 mm] vs 0.10 mm [0.08–0.11 mm]; *P *< .001).

In accordance with the improved efficiency, the median contouring time per lesion was significantly shorter with than without BMSS assistance (140 s [131–149 s] vs 75 s [70–79 s]; *P* < .001), with a median time saving of 42% (40–45%). Resident readers gained a similar contouring time saving as attending readers (reduced median time, 44% [40–47%] vs 40% [37–44%]; *P* = .92; Figure 5C). However, the time-saving benefit had an inhomogeneous effect on efficiency improvement among readers (median time-saving range, –15% to 99%). There was a significant difference in contouring time saving between the first and the second sessions (46% [43–49%] vs 39% [36–41%]; *P* = .001). Delineation of the median time savings at the patient level showed a similar trend as the time saving at the lesion level (all readers, 36% [32–40%], *P* < .001; resident, 37% [31–43%], *P* < .001; attending, 35% [31–40%]; *P* < .001; resident vs attending, 38% [31–43%] vs 36% [31–40%], *P* = .813). Spearman’s correlation analysis showed that the time saving was correlated with tumor volume (*r* = 0.049 [0.005–0.092], *P *= .029). However, no correlation was found between tumor diameter and time saving (*r* = 0.033 [−0.011 to 0.076], *P *= .140). Overall, when assisted, the DSC, ASD, sensitivity, specificity, and MCC were positively correlated with the contouring time (all *P* < .05), while the RVD was negatively correlated with the contouring time (Supplementary Figure 5 and Table 3).

Consistent with the improvement in DSC, the RVD was significantly reduced by 53% (median assisted RVD vs median unassisted RVD, 9% [9–10%] vs 19% [18–20%], *P* < .001) when all readers were included in the analysis. Similar to the improvement in the DSC, the reduction in the median assisted RVD was higher for junior readers than for attending radiologists (12.4% vs 7.7%, *P *< .001). Further, we graded the volume that needed to be corrected, and the trends in RVD values pre- and post-assistance are shown in Figure 5D. Representative examples of segmentation are provided in Supplementary Materials Part 6.

## Discussion

AI models for BM auto-segmentation have been reported, but most previous studies have been preclinical and have evaluated the standalone performance of AI.^11,15–17,25^ In this multi-center, prospective, randomized crossover multi-reader evaluation study, the proposed model exhibited high, robust segmentation performance with a DSC of 0.91 and an ASD of 0.40 mm for the multi-center dataset. The reading experiment demonstrated that BMSS assistance significantly improved BM segmentation and resulted in a 42% reduction in the number of working hours for physicians with different levels of experience. The size and diversity of this multi-center dataset have helped provide insights into the performance of deep learning in a virtual clinical setting. Our study may serve as a blueprint for the application of AI in BM segmentation to improve SRS practice.

SRS for brain metastases is labor-intensive and requires manual target contour generation, resulting in operator dependence and variability. For manual target delineation, the accuracy and working time are a pair of contradictions in clinical practice, where the trade-offs between them can be tricky for doctors. AI can serve as a useful tool for balancing such challenge by improving accuracy while reducing time. Operator variations are also worthy of consideration. A study investigating target delineation at 22 SRS centers showed that inter-observer contouring variations were expected to be exacerbated for small metastases.^6^ This operator variability has been identified as a significant factor in the quality assurance of brain tumor SRS.^6,8^ To address these issues, a substantial number of studies have been devoted to developing techniques for the automatic segmentation of BMs. These techniques have reported DSC values of 0.77–0.85.^13,15,17^ While most previous studies have lacked external and clinical validation and, as such, are proof-of-concept studies,^26^ our study provides a more meaningful clinical evaluation of the standalone performance of AI by incorporating a reading experiment. In the prospective multi-center dataset, our algorithm resulted in higher DSC values for segmenting BMs than the algorithms used in previous studies. This result is credited to the large number of lesions in the dataset, the use of a manually annotated reference, and the diverse images derived from various acquisition protocols captured using a large variety of scanners. Moreover, our data may be representative of the clinical primary tumor spectrum, as they encompass 32 types of extracranial primary tumors. Consequently, the performance was not dependent on the scanner manufacturer, acquisition protocol, or primary tumor classification. The generalizability of the BMSS was supported by validation using an external dataset from 4 centers, with similar segmentation results between the internal and external datasets.

Despite recent advances in AI for BM segmentation, there is a large gap between proof-of-concept and robust, prospectively validated algorithms. Most previous studies have been proof-of-concept studies. A thorough validated investigation provides important information on the feasibility of the clinical implementation of AI models. An MRMC study is recommended for such an investigation. A randomized crossover reading study reduces the memory bias caused by sequential reading and thus is a preferable reading design.^27^ Our segmentation experiment showed that BMSS assistance significantly augmented radiologists’ performance by increasing segmentation accuracy and inter-reader agreement and decreasing the number of working hours compared with unassisted reading. Specifically, we found that the accuracy improvement was greater for junior radiologists than senior radiologists, but the time-saving benefit was similar. These results were also partly supported by a single-center study,^25^ which is the only other study to clinically validate BM segmentation. In that study, Lu et al. combined DeepMedic and 3D U-Net to segment brain tumors. They validated their model using a reading study involving a limited dataset of 10 cases with 3 types of tumors, attaining an overall DSC value of 0.84. They found that non-SRS specialists also gained greater contouring accuracy improvement, but less time reduction, than SRS specialists when assisted by the model. They also found that the DSC value and time improvements were not related to the reading sequence. Although contouring improvements appeared to differ between specialists, the DSC value improvements were not different (both 0.04 in the 2 reading modes), and the unassisted-first mode had a greater time saving than the assisted-first mode. The possible reason for these results was that there was inevitable memory bias, despite the randomized crossover design, and the sample size of their study may have been too small to allow clinically meaningful estimations of the performance metrics.

DSC is a widely accepted index for evaluating the accuracy of image segmentation. However, it is not always accurate and meaningful when assessing performance on large lesions. There is an inherent limitation of this metric as it increases with lesion volume, indicating that it is sensitive to small lesions.^28^ We noted that our algorithm performed less well at delineating margins for some large lesions, despite obtaining a high DSC value. For clinically relevant assessments, we provided additional metrics for the BMSS and the readers. Therefore, we conducted correlation and consistency analyses of the PV and GV and used ASD as a supplementary index to assess the surface coincidence between the AI-generated and reference contours. Given that the DSC index does not provide insights into how much the AI-generated contours need to be revised to be used in clinical practice, we computed the volume difference between AI-generated and expert-established contours, designated as the RVD, to reflect volumetric revision.

In previous studies, AI initially improved the detection and segmentation of BM. We hypothesized that it may also have the potential to optimize radiotherapy practice related to BMs. Radiotherapy dose and fractionation regimens for BMs mainly depend on the type of primary tumor, brain metastasis characteristics (the number, size, and location), adjacent organs at risk, and the radiation techniques used.^4,5^ Therefore, it is essential to exactly position and accurately quantify lesions. Precision radiotherapy requires a precise radiation range and dose to avoid insufficient tumor coverage and excessive exposure to surrounding tissues. A high degree of geometric and dosimetric accuracy is critical. In multiple SRS procedures for unresectable BMs, the gross tumor volume is significantly correlated with the local control rate.^29^ A straightforward way to ensure coverage is to extend the margin of the gross tumor. However, extended boundaries increase the radiation dose to normal brain tissue and increase the risk of radiation-induced brain damage. Noel et al. compared 0- and 1-mm margins and found that a 1-mm margin improved local control without a significant increase in toxicity.^30^ The BMSS reduced the ASD and improved the specificity compared with unassisted segmentation, indicating that BMSS-assisted margin delineation had a higher degree of agreement with the GT and thus may be able to enhance geometric accuracy. Our series of studies of detection and segmentation provide valuable evidence for the clinical diagnosis and treatment of BMs. Whether such advances benefit patients, for example, by improving local control and reducing radiotoxicity, remains an area worth studying.

Despite the model performing well and appearing to surpass human performance at BM segmentation, when evaluated with summary statistics using prospective data, the model appeared to be prone to errors and underperformed for BMs that humans would consider easy to identify. The accuracy of our model was reduced when segmenting specific lesions (Supplementary Figure 7). The prediction mask for areas with a sharp signal change was in poor agreement with the reference standard. Typically, lesions with low or very low signal are excluded by AI tools. A possible explanation for this limitation is that the recognition of lesions by AI is based on a certain gray threshold, and lesions usually show high enhancement; thus, hypo-intensive areas are not recognized as abnormal. Lower DSC values were generated for the segmentation of irregular lesions rather than for the segmentation of lesions with a regular shape. We speculate that the BMSS may not be sufficiently sensitive to capture drastic changes at the edges of the lesion. Another finding was the unstable segmentation performance of lesions adhering to the meninges. It is uncertain whether both lesions close to the meninges and the adjacent meninges were included in the prediction mask. In clinical practice, delineating such lesions is also challenging for physicians, and they present greater operator variability than lesions within the brain parenchyma. The AI-derived volume should be interpreted with caution due to the known failings of AI when applied to such cases. The abovementioned issues should be addressed during arithmetic optimization.

There are some limitations of our study. First, the constructed network is currently limited to BMs. Applications extending to multiple classes of brain tumors, such as glioma, meningioma, and acoustic neuroma, should be explored in future studies. Second, although a relatively large number of radiologists with different qualification levels participated in the reading experiment, they were all recruited from a single cancer referral center. Therefore, it is not clear whether this model is feasible for use in general hospitals. Third, given that gradient echo is routinely adopted for brain 3D contrast imaging in our center, the proposed network was trained almost entirely on such sequences, but the current model was optimized using data acquired by fast-spin echo. Fourth, in the prospective evaluation, considering the application of SRS to patients with limited metastases and the reader workload, we restricted the enrolment of patients. In addition, the sample size of the test set was much smaller than that of the training set, and the tumor size was also larger in the test set. This imbalance among datasets should be considered when interpreting the findings.

## Conclusions

Overall, the proposed fully connected CNN may be utilized to automatically, accurately, and stably segment BMs. AI assistance improved the accuracy and efficiency of the manual delineation of BMs. The integration of a state-of-the-art AI tool into current workflows has the potential to optimize SRS schemes and improve patient care.

## Supplementary material

Supplementary material is available online at *Neuro-Oncology* (https://academic.oup.com/neuro-oncology).

noae113_suppl_Supplementary_Material

## Data Availability

The raw image data used to develop the AI model are not shareable, in accordance with institutional requirements governing human subject privacy protection. Data generated by the authors or analyzed during the study are available in the Research Data Deposit public platform at www.researchdata.org.cn (RDD no. RDDB2022116272). The algorithm code will be made available immediately following publication to anyone who wishes to access the data. Requests for access to the code can be sent to the corresponding author.

## Notes

This paper being submitted is the third in a series of artificial intelligence for brain metastases; the first 2 have been published in *Neuro-Oncology*.
