# Supplementary material for: Automated segmentation of brain metastases with deep learning: A multi-center, randomized crossover, multi-reader evaluation study
Source: Neuro Oncol. 2024 Jul 11;26(11):2140–51. doi: 10.1093/neuonc/noae113 (PMC11639187; doi:10.1093/neuonc/noae113)
Supplement: noae113_suppl_Supplementary_Material [file noae113_suppl_supplementary_material.docx]

**Supplementary Materials**

**Part 1. The inclusion and exclusion criteria of the development set and the test set**

We included patients (1) with extracranial primary tumour(s) confirmed by pathology; (2) with newly developed brain metastases (BMs); and (3) who underwent 3D-enhanced brain MRI and had at least one follow-up MRI. Only the initial scan with at least one lesion was assessed in this study. We excluded patients (1) with primary intracranial tumour(s); (2) with leptomeningeal metastasis; (3) who had undergone brain surgery; or (4) with excessive artifacts in their images. There were no limitations on the sizes of the metastases. Four hundred and ninety-seven patients included in the current study for development set were reported in a prior study^1^. The prior report evaluated deep learning model on brain metastasis detection. The current study focuses on lesion segmentation, expands on prior study patients by collecting new multi-center patients for testing, and includes new analyses of algorithm performance and aided segmenting study. Given the workload in the reading experiment, patients in the test set with more than 15 lesions were excluded.

From 15 August 2019 to 18 November 2020, 624 consecutive patients with newly diagnosed brain metastases at Sun Yat-sen University Cancer Center were retrospectively included in the development set. We excluded 136 patients for the following reasons: (1) the presence of a primary intracranial tumour (n = 22); (2) the presence of parenchymal BMs involving the leptomeninge, or leptomeningeal metastases alone (n = 69); (3) a history of surgery (n = 21); or (4) excessive artifacts in images (n = 24). Final data from 488 patients with 10,338 BMs were used to develop the network.

The prospective test set was collected from five medical centres, Sun Yat-sen University Cancer Center, Meizhou People’s Hospital, Dongguan People’s Hospital, Fujian Cancer Hospital, and The First Affiliated Hospital of Guanzhou Medical University, each of which eventually enrolled ten consecutive eligible participants with a total of 203 BMs. The details are shown in Table S1.

| **Table S1 Included and excluded participants in the prospective test set** | | | | | |
| --- | --- | --- | --- | --- | --- |
| **Institutions** | **Date** | **Included** | **Excluded** | **Eligible** |  |
| SYSUCC | 2021.2.8- 2021.3.11 | n = 26 | ① intracranial primary tumour (n = 2)  ② history of brain surgery (n = 1)  ③ leptomeningeal metastasis (n = 5)  ④ number of metastases > 15 (n = 3)  ⑤ obvious artifacts (n = 1) | n = 14 |  |
| MZPH | 2021.2.9- 2021.3.21 | n = 25 | ① intracranial primary tumour (n = 1)  ② history of brain surgery (n = 1)  ③ leptomeningeal metastasis (n = 2)  ④ number of metastases > 15 (n = 4)  ⑤ obvious artifacts (n = 3) | n = 14 |  |
| DGPH | 2021.2.4- 2021.4.9 | n = 28 | ① history of brain surgery (n = 1)  ② leptomeningeal metastasis (n = 2)  ③ number of metastases > 15 (n = 4)  ④ obvious artifacts (n = 5) | n = 16 |  |
| FJCH | 2021.2.4- 2021.3.30 | n = 35 | ① intracranial primary tumour (n = 2）  ② history of brain surgery (n = 2)  ② leptomeningeal metastasis (n = 4)  ③ number of metastases > 15 (n = 5)  ④ obvious artifacts (n = 3) | n = 19 |  |
| GZFH | 2021.7.13-2021.8.30 | n = 27 | ① intracranial primary tumour (n = 2)  ② history of brain surgery (n = 2)  ③ leptomeningeal metastasis (n = 3)  ④ number of metastases > 15 (n = 6)  ⑤ obvious artifacts (n = 3) | n = 11 |  |

SYSUCC, Sun Yat-sen University Cancer Center; MZPH, Meizhou People’s Hospital; DGPH, Dongguan People’s Hospital; FJCH, Fujian Cancer Hospital; GZFH, The First Affiliated Hospital of Guanzhou Medical University; n, the number of participants

**Part 2. MRI scanning protocols**

**2.1 Development set and test set from Sun Yat-sen University Cancer Center**

MRI was performed using a 1.5 T (MAGNETOM Aera, Siemens, Munich, Germany; Achieva, Philips, Amsterdam, Netherlands) or 3.0 T (Discovery MR750, MR750W, SIGNA Pioneer, SIGNA Premier, or SIGNA Architect, GE Healthcare, Chicago, IL, USA; Ingenia CX, Philips; MAGNETOM Trio Tim or MAGNETOM Prisma, Siemens; uMR560, uMR780, or uMR790, United Imaging, Shanghai, China) scanner with an 8-, 16-, 32-, or 64-channel head coil. Three-dimensional contrast-enhanced T1-weighted imaging (3D T1WI) was performed after intravenous injection of a contrast material (MultiHance, Bracco Diagnostics, Princeton, NJ, USA, 0.1 mmol/kg; Gadobutrol or Gadovist, Bayer Schering Pharma AG, Berlin, Germany, 0.1 mmol/kg), followed by a 15 mL saline flush with an injection velocity of 2.0 mL/s.

**2.2 Test set from Meizhou People’s Hospital**

All patients underwent MRI scans on a 1.5 T (Optima MR360, GE Healthcare) or 3.0 T (MAGNETOM Skyra, MAGNETOM Prisma, Siemens) scanner using a 16-channel head coil. The axial brain volume sequence of the BRAVO or MPRAGE sequence was performed on the patients 5 minutes after the administration of 0.1 mmol/kg body weight of contrast agent (Gd-DTPA, Magnevist; Bayer Schering Pharma AG), followed by a 15 mL saline flush with an injection velocity of 2.0 mL/s.

**2.3 Test set from Dongguan People’s Hospital**

All patients underwent MRI scans on a 3.0 T scanner (MAGNETOM Skyra or MAGNETOM Verio, Siemens) using a 16-channel head coil. 3D CET1WI was performed on all patients 5 minutes after the administration of 0.1 mmol/kg body weight of contrast agent (gadoxetic acid disodium, Primovist, Bayer Vital GmbH, Leverkusen, Germany), followed by a 20 mL saline flush with an injection velocity of 2.0 mL/s.

**2.4 Test set from Fujian Cancer Hospital**

Patients underwent MRI scans on a 3.0 T scanner (Discovery MR750W) using a 16-channel head coil. 3D CET1WI was also performed on all patients 5 minutes after the administration of 0.1 mmol/kg body weight of contrast agent (gadoxetic acid disodium), followed by a 15 mL saline flush with an injection velocity of 2.0 mL/s.

**2.5 Test set from The First Affiliated Hospital of Guanzhou Medical University**

Patients underwent MRI scans on a 1.5 T (Achieva) or 3.0 T scanner (Ingenia CX) using a 16-channel head coil. 3D CET1WI was also performed on all patients 5 minutes after the administration of 0.1 mmol/kg body weight of contrast agent (gadoxetic acid disodium), followed by a 15 mL saline flush with an injection velocity of 2.0 mL/s.

The typical imaging parameters of all participating institutions are shown in Table S2.

| **Table S2 Key parameters of 3D CET1WI sequences across five centers** | | | | | |
| --- | --- | --- | --- | --- | --- |
| **Parameters** | **Institutions** | | | | |
| Technique | SYSUCC | MZPH | DGPH | FJCH | GZFH |
| TR (msec) | 1,660–2,300*^a^* | 2,300/2,300/12.3*^b^* | 2,200/1,900 | 7.00 | 6.6/8.2*^b^* |
| TE (msec) | 2.07–3.0*^a^* | 2.26/2.28/5.1*^b^* | 2.26/2.45 | 2.53 | 3.0/3.7*^b^* |
| Flip angle (degrees) | 8, 9 | 8, 8, 12*^b^* | 9 | 9 | 8*^b^* |
| FOV (mm^2^) | 256×256, 240×240 | 256×256, 250×250, 240×240*^b^* | 256×256, 250×250 | 256×256 | 240×240*^b^* |
| Acquisition matrix | 256×256, 240×240 | 512×512, 250×250, 240×240*^b^* | 256×256, 250×250 | 256×256 | 240×240*^b^* |
| Voxel size | 1×1×1 | 0.5×0.5×0.5, 1×1×1, 1×1×1*^b^* | 1×1×1 | 1×1×1 | 1×1×1*^b^* |
| Slice thickness | 1 | 1 | 1 | 1 | 1*^b^* |
| Number of excitations | 2 | 1 | 1 | 1 | 1*^b^* |
| Acquisition plane | Sagittal | Sagittal/Axial*^b^* | Sagittal | Sagittal | Sagittal |

^a^ range for various scanners, *^b^* for 1.5T scanners

3D CET1WI, three-dimensional contrast-enhanced T1-weighted imaging; TR, repetition time; TE, echo time; FOV, field of view; SYSUCC, Sun Yat-sen University Cancer Center; MZPH, Meizhou People’s Hospital; DGPH, Dongguan People’s Hospital; FJCH, Fujian Cancer Hospital; GZFH, The First Affiliated Hospital of Guanzhou Medical University

**Part 3. Brain metastasis segmentation model**

The Brain metastases segmentation system (BMSS) was modified based on V-Net and designed as VB-Net^2^. The main goal was to apply model compression to reduce the extensive number of network parameters and allow the rapid training and deployment of the segmentation network. Specifically, we added a bottleneck structure to V-Net to form VB-Net. Each down-block and up-block was replaced with the bottleneck structure. The bottleneck included three convolutional layers. The first layer reduced the number of channels using a 1×1×1 convolution kernel, the second layer performed a 3×3×3 spatial convolution, and the third layer applied a 1×1×1 convolution kernel to increase the number of channels to match the V-Net structure. Thus, the number of parameters was significantly reduced, and performed as well as the original network. Finally, without loss of accuracy, the size of the VB-Net model was reduced by 96.5% compared to V-Net.

Model implementation was based on the Pytorch framework, an open-source Python deep-learning library^3^. We used focal loss (α = 2; β = 0.999) as the loss function. The model was trained using the Adam optimisation algorithm to dynamically adjust the learning rate. The initial learning rate was 1 × 10^−4^. The training used an Intel® Xeon® CPU E5-2698 v4 @ 2.20 GHz central processing unit and an Nvidia Tesla V100-SXM2, 32G × 8 graphics processing unit with CUDA version 10.1. Additionally, data augmentation was performed to enrich the training dataset.

The segmentation network was described as follows.

**3.1 Lesion segmentation algorithm**

**3.1.1 VB-Net**

Detected lesions were automatically segmented using an end-to-end 3D segmentation model derived from V-Net, known as VB-Net proposed in a previous study^2^. The main goal was to apply model compression to reduce the large number of network parameters and to be able to quickly train and deploy the segmentation network. For example, each 3D V-Net model occupies 250 MB. In brain metastasis screening, multiple segmentation networks are needed to form a complete workflow, and it would be challenging to develop an efficient model if the segmentation networks were not tailored. A large amount of parameter redundancy also results in wasted storage space and low computational efficiency, which ultimately leads to difficulties in the promotion and use of the model.

Specifically, we added a bottleneck structure to the V-Net structure to form VB-Net, as shown in Figure S1. Each down-block and up-block was replaced with the bottleneck structure. The bottleneck included three convolutional layers. The first layer reduced the number of channels using a 1×1×1 convolution kernel, the second layer performed a 3×3×3 spatial convolution, and the third layer applied a 1×1×1 convolution kernel to increase the number of channels to match the V-Net structure. In this way, the number of parameters was significantly reduced, and the experimental results showed that the new network performed as well as the original network. Finally, without loss of accuracy, the size of the VB-Net model was reduced to 8.8 MB from the original 250 MB of V-Net. This makes the VB-Net model suitable for many common purposes, including use on mobile devices.


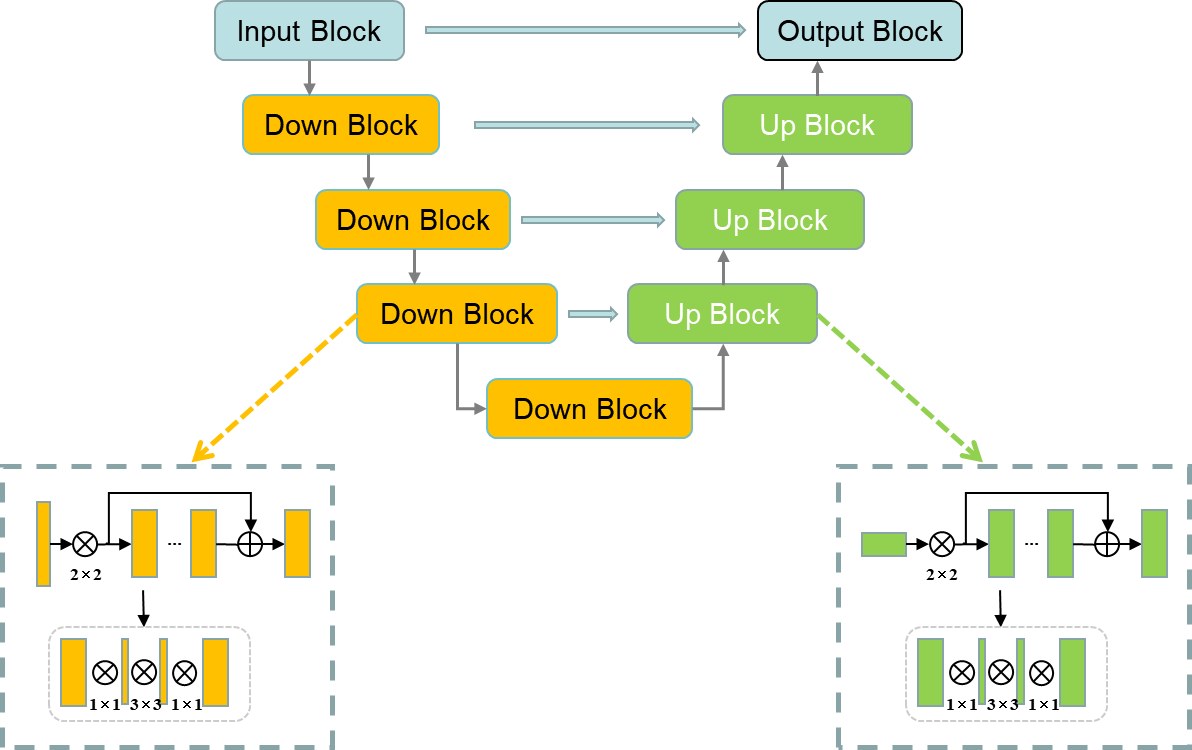


**Figure S1 The structure of VB-Net**

**3.1.2 Loss functions**

Loss functions play an important role in determining segmentation performance. Focal loss introduces the parameter γ, relative to cross-entropy loss, to reduce the weight of easy-to-classify samples so that the network pays more attention to difficult samples. The Dice similarity coefficient is often used as the evaluation metric, but it should be considered as the optimisation index. Combining the advantages and disadvantages of the above two loss functions, we propose calculation of the double loss as follows:

$double loss=a\times focal loss+b\times Dice loss$,

where ***a*** and ***b*** are the weights for focal loss and Dice loss, respectively, and ***a + b = 1.***

**3.1.3 Metastasis segmentation**

Due to the inconsistent sizes of brain metastases, which may range from 3 mm to 50 mm, a fixed-box brain metastasis segmentation method was proposed, as shown in Figure S2. This method performed fine segmentation of brain metastases based on the detection results or by manually labelling the detection location information of brain metastases.

**Figure S2 The training process of the segmentation model**

VB-Net

Fixed-box resample

Fine-resolution model

Normalise


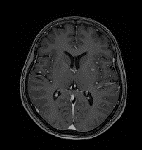

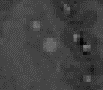

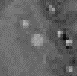

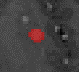


**3.2 Training**

First, to solve the problem of the inconsistent size of metastases, we proposed a fixed-box sampling method. Brain metastases were specifically cropped from the original MR image at 2.5 times the size of the detection box, and then the size of the input image was uniformly resampled to a fixed size (such as 96×96×96). As shown in Figure S3, the same image size allowed brain metastases of different sizes to be completely included and the proportions in the image were the same. The normalised image brightness value within the specified range, such as [- 1,1], was used to accelerate the convergence of the model, and train the fine-resolution model.

**Figure S3 Images of brain metastases of different sizes after using the fixed-box sampling method**


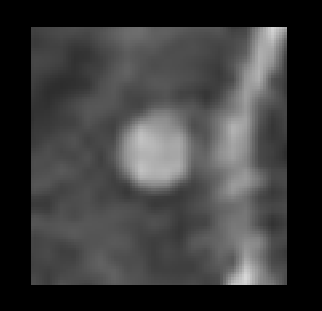

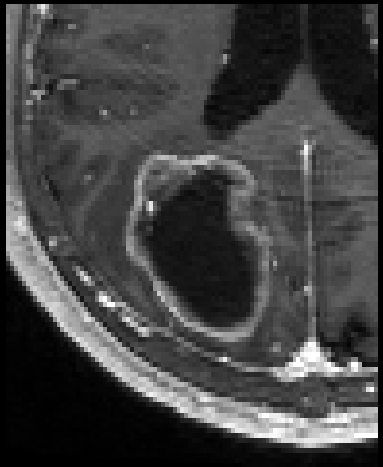


**3.3 Validating**

During the test (Figure S4), brain metastases were cropped from the original MR image using the same method as the fixed box. After normalization, the image was input into the trained segmentation network to obtain the segmentation results, which were resampled to the size of the original image to obtain the final segmentation results of the brain metastases.

Fine-resolution model

Fixed-box resample

Normalise

Resample


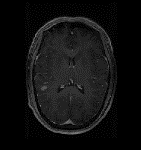

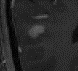

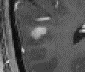

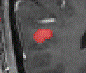

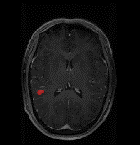


**Figure S4 The validating process of the segmentation model**

**3.4 Summary**

This study focused on the segmentation of brain metastases using segmentation network. After segmenting the brain region, metastasis detection and lesion segmentation and measurement were performed. A multi-scale brain metastasis detection network was employed and improved by using multi-resolution, multi-head detection and a cascade structure for detecting lesions with different shapes and sizes. The multiple-layer cascade network helped reduce the number of false negatives while maintaining high sensitivity. Finally, a bagging classification network was applied to further refine the detection network. In lesion segmentation, an end-to-end image segmentation network (VB-Net) was developed, which applies a bottleneck network for model compression, and finally, accurate quantification was achieved to assist lesion measurement (Figure S5).

**Functional area segmentation**

**Skull stripping**

**MRI images**

**Lesion detection**

**Lesion location**

**Lesion segmentation**


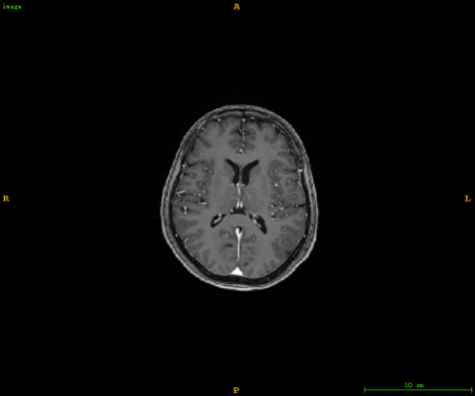

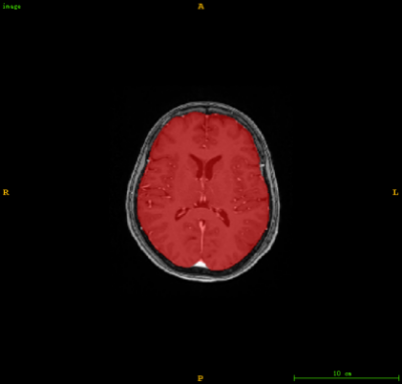

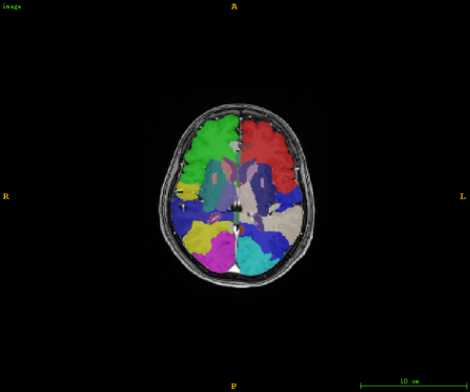

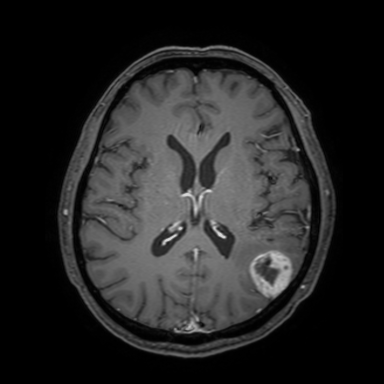

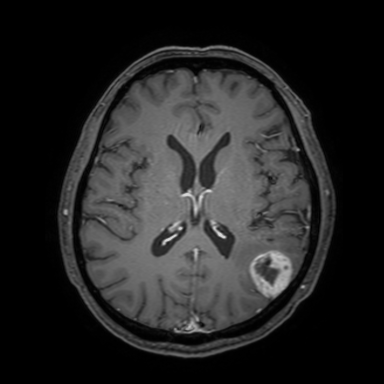


**Figure S5 Algorithm framework of assisted brain metastasis detection and segmentation**

**Part 4. Sample size calculation for the test set and definition for patient-wise DSC, voxiel-wise sensitivity, specificity and Matthews correlation coefficient**

**4.1** The sample size required for the test set was calculated according to the results obtained in our preliminary segmenting experiment and the assumption that the Dice similarity coefficient (DSC) of the reader is better with than without BMSS assistance. Therefore, a one-sided superiority test was performed. The following sample calculation was used (4):

$\text{n}_{T}=\text{n}_{\text{C}}=\frac{(Z_{1\text{-}\partial}+Z_{1\text{-}\beta})^{2}\left[DSC_{C}(1-DSC_{C})+DSC_{T}(1-DSC_{T})\right]}{(\left|D\right|-\Delta)^{2}}$.

DSC_T_ and DSC_C_ are the expected DSCs of AI-assisted and unassisted joint segmentation, respectively. According to the DSC value for the model using the internal test set, the model segmentation DSC_M_ value was predicted to be 0.85. Five patients with fewer than ten brain metastases and a total of 28 nodules were randomly selected from the test set. Two residents and two attending radiologists delineated the tumour contours independently, and the median segmentation DSC_R_ value for the four doctors was 0.8. The lowest expected DSC_T_ value for AI-assisted segmentation was 0.85.

|D| is the absolute value of the expected difference between DSC_T_ and DSC_C_, |D| = |DSC_T_-DSC_C_ | = 0.85–0.8 = 0.05. △ is the superiority boundary value, and the positive value was taken.

The test power was set as 1-*β* = 0.8, with a significance level of *α* = 0.025 (one-sided), Z_1-_*_α_* = 1.64, and Z_1-_*_β_* = 1.28. A minimum number of 50 brain metastases per group was calculated to be required.

If the sample size ratio of AI-assisted and unassisted segmentation was 1:1, at least 100 brain metastases would be required. Only patients with ≤15 metastases were included in the multi-reader, multi-case study, and the sampling study performed at our centre showed that the average number of metastases per patient was expected to be four. Therefore, n = 100/4 = 25 patients were required to be enrolled, and approximately five patients were required to be enrolled from each of the five centres participating in the test set. Assuming an expected dropout rate of 20%, at least 32 patients should be initially enrolled from the five centres. Finally, we consecutively included ten eligible participants from each centre.

**4.2** Equal weight is assigned to each lesion in a patient with multiple metastases. The patient-wise DSC_p_ can be calculated as:

$${DSC}_{p}=\frac{{DSC}_{1} +{DSC}_{2}+{DSC}_{3} \cdots+\cdots{DSC}_{n}}{n}$$

Where n is the metastasis number of the patient; $\frac{1}{n}$ represents the weight of each lesion.

**4.3** Voxel-wise sensitivity, specificity and MCC are defined as follows^6,7^.

$$Sensitivity =\frac{TP}{TP +FN}$$

$$Specificity =\frac{TN}{TN +FP}$$

$$MCC =\frac{TP \times TN - FP\times FN}{\sqrt{(TP + FP)(TP + FN)(TN + FN)(TN + FP)}}$$

Where TP = true positives, FP = false positives, TN = true negatives, and FN = false negatives.

**Part 5.** **The sensitivity, specificity and MCC of readers; correlation between assisted performance and contouring time**

**
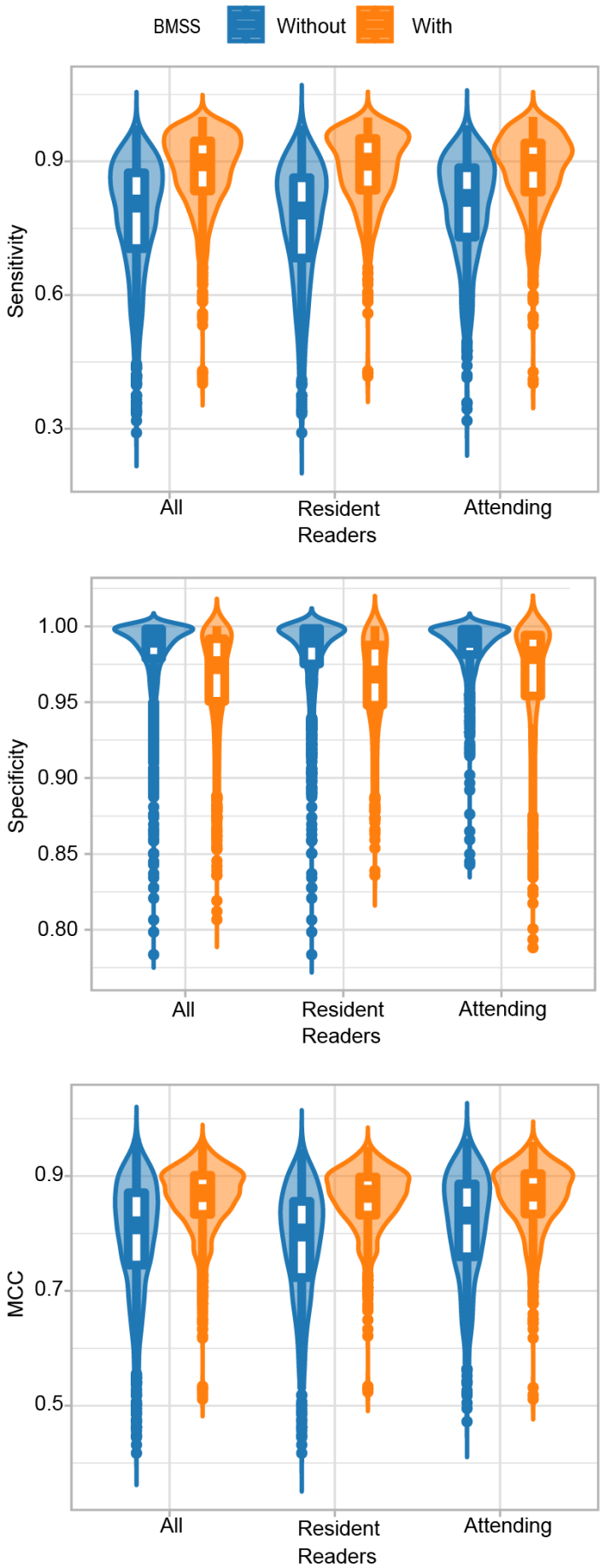
Figure S5 The sensitivity, specificity and MCC of reader with and without BMSS**

**Table S3 Correlation between** **assisted-reader performances and contouring time**

| Performance | Spearman rho ^a^ | | |  | *P* | | |
| --- | --- | --- | --- | --- | --- | --- | --- |
|  | All | Resident | Attending |  | All | Resident | Attending |
| DSC | 0.125＊ | 0.101＊ | 0.171＊ |  | < 0.001 | 0.001 | < 0.001 |
| ASD | 0.284＊ | 0.292＊ | 0.281＊ |  | < 0.001 | < 0.001 | < 0.001 |
| RVD | -0.045＊ | -0.065＊ | -0.056 |  | 0.045 | 0.038 | 0.073 |
| Sensitivity | 0.128＊ | 0.147＊ | 0.145＊ |  | < 0.001 | < 0.001 | < 0.001 |
| Specificity | 0.045＊ | -0.055 | 0.080＊ |  | 0.044 | 0.083 | 0.010 |
| MCC | 0.174＊ | 0.115＊ | 0.236＊ |  | < 0.001 | < 0.001 | < 0.0001 |

a, correlation tested by Spearman rank correlation; ＊significant

DSC, dice similarity coefficient; ASD, average surface distance, MCC, Matthews Correlation Coefficient, RVD, relative volume difference

**Part 6. The representative examples of model and readers segmentation**

**
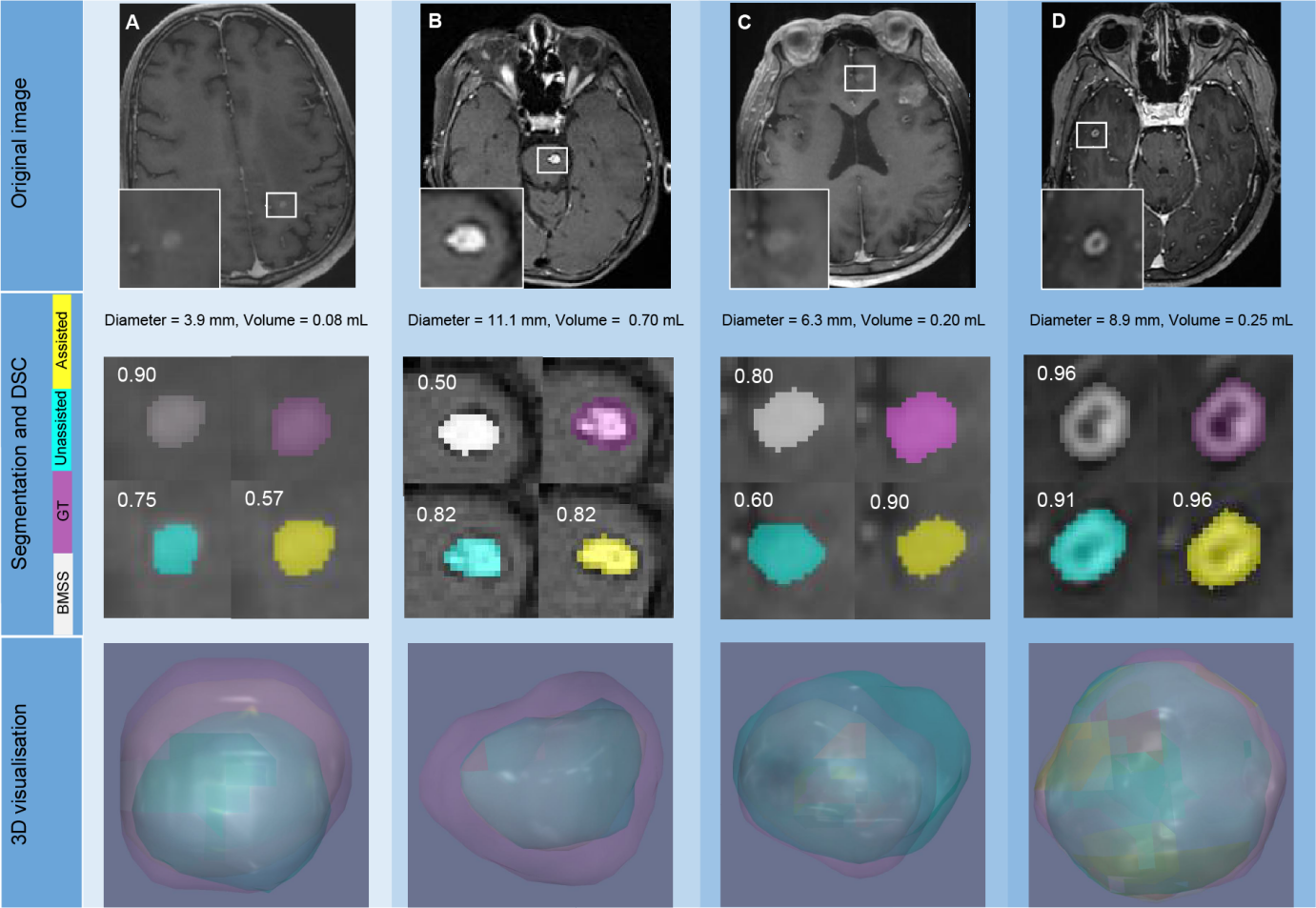
**

**Figure S7 The representative examples of model and readers segmentation**

(A) the smallest lesion in the test set, and (B) the lowest, (C) middle, and (D) highest DSC values obtained with the model.

BMSS, brain metastases segmentation system; DSC, dice similarity coefficient; ASD, average surface distance; GT, ground truth.

**References**

1. Yin S, Luo X, Yang Y, et al. Development and validation of a deep-learning model for detecting brain metastases on 3D post-contrast MRI: a multi-center multi-reader evaluation study. Neuro Oncol 2022; 24(9): 1559-70.
2. Han M, Zhang Y, Zhou Q, et al. Large-scale Evaluation of V-Net for Organ Segmentation in Image Guided Radiation Therapy. Conference on Medical Imaging - Image-Guided Procedures, Robotic Interventions, and Modeling; Feb 17-19; San Diego, CA; 2019.
3. PyTorch. *https://pytorch.org/*. Published 2021. Accessed 2021 October 28.
4. Deng W, He J. Statistical description and statistical inference of quantitative data. In: Deng W, ed. Design and Statistical Analysis of Clinical Trials. Beijing, People's Medical Publishing House, 2012; 120-125.
5. Yousefi S, Kehtarnavaz N, Gholipour A. Improved labeling of subcortical brain structures in atlas-based segmentation of magnetic resonance images. IEEE Trans Biomed Eng 2012;59(7):1808-1817..
6. Taha AA, Hanbury A. Metrics for evaluating 3D medical image segmentation: analysis, selection, and tool. BMC medical imaging. 2015; 15(1):29.
7. Chicco D, Jurman G. The Matthews correlation coefficient (MCC) should replace the ROC AUC as the standard metric for assessing binary classification. BioData mining. 2023; 16(1):4.
